# Neurobiology and Impact of Chronic Traumatic Encephalopathy in Athletes: A Focused Review

**DOI:** 10.7759/cureus.86367

**Published:** 2025-06-19

**Authors:** Jenny T Makhoul, Anastasia G Nasr, Zemar G Qazi, Brian J Piper, Areeba N Ahmed, Amna Javeed

**Affiliations:** 1 Medical Education, Geisinger Commonwealth School of Medicine, Scranton, USA

**Keywords:** cerebrospinal fluid (csf), chronic traumatic encephalopathy (cte), mild traumatic brain injury (mtbi), neurodegeneration, phosphorylated transactive response dna-binding protein 43, positron emission tomography, repetitive mild traumatic brain injury, tau protein

## Abstract

Recent reports have highlighted a troubling pattern of athletes exhibiting pronounced and unexplained behavioral changes. This phenomenon is often linked to chronic traumatic encephalopathy (CTE), a progressive neurodegenerative disorder associated with repeated head injuries in contact sports, and is characterized by the accumulation of abnormal tau protein in the brain. This paper provides a focused review of the neurological and neurobehavioral mechanisms, prevention and treatments, and the role of animal models in advancing our understanding of CTE. Key findings have identified the accumulation of tau proteins and neuroinflammation as major contributing factors in the development of CTE. However, gaps in the literature, including the need for standardized injury models and biomarkers for diagnosing CTE in living individuals, point to future directions in refining diagnostic tools and developing targeted therapeutic interventions.

## Introduction and background

Chronic traumatic encephalopathy (CTE) is a progressive neurodegenerative disorder associated with repeated head injuries (RHIs), mostly seen in athletes participating in contact sports [1]. Today, CTE is recognized not only in professional athletes but also in teens and amateur athletes, raising pronounced public health concerns [1-3]. Notably, neuropathological evidence of CTE has been identified in individuals as young as 17 years old, highlighting the risks of repetitive head impacts even at an early age [1].

CTE was first described in 1928 by Dr. Harrison Martland as punch drunk syndrome in a group of boxers. CTE has since been reported to present with symptoms such as memory loss, cognitive decline, behavioral changes (e.g., aggression or impulsivity), mood disturbances (e.g., depression), speech and motor abnormalities, and in later stages, dementia [1,4,5]. However, it was not until Dr. Bennet Omalu, physician and forensic pathologist, published evidence in 2002 of CTE in former National Football League (NFL) player Mike Webster, who had passed away at age 50 after experiencing cognitive impairment, mood changes, and Parkinsonian symptoms - that this condition gained attention and was named [6]. CTE is associated with, but not limited to, sports such as American football, ice hockey, rugby, and wrestling, where athletes are exposed to frequent and repeated head impacts [1,2,7]. However, despite increasing awareness of CTE in athletes, female athletes remain significantly underrepresented in research, raising concerns about how sex-specific factors may influence the development, diagnosis, and progression of the disease [8]. This disorder progression can lead to severe neurological conditions over time, including worsening cognitive decline, mood disturbances, and behavioral changes [1,2,7,9]. These symptoms commonly emerge 8 to 10 years following repeated mild traumatic brain injuries (mTBIs) [10]. An epidemiological study has shown that more than 40% of deceased individuals with a history of contact sports have CTE [11]. Currently, CTE can only be diagnosed by examining brain tissue once deceased [11,12]. Despite significant advancements, there are currently no effective therapeutic options available to combat this progressive disorder, highlighting the urgent need for continued research into effective treatments and preventive measures [13]. Research efforts are focused on understanding the types of head impacts that carry the highest risk of developing the disorder, improving diagnostic methods, and further characterizing neurobiological mechanisms underlying CTE [14]. Additionally, addressing CTE involves preventative strategies, including modifications in sports practices and regulations aimed at reducing head injuries and protecting athletes [15,16].

As this is a narrative review, we did not follow a formal systematic methodology. Instead, we selected recent and relevant literature to provide a focused synthesis of current findings and highlight critical gaps in CTE research. This review explores the symptoms and diagnosis of CTE, neurobehavioral and neurobiological mechanisms, prevention and treatment strategies, and the use of mouse models in advancing our understanding of the disease. Current pathological insights include the accumulation of phosphorylated tau and transactive response DNA-binding protein 43 kDa (TDP-43), along with chronic neuroinflammation contributing to neurodegeneration. While no disease-modifying treatments are currently available, recent preclinical and clinical studies are investigating anti-inflammatory and tau-targeting therapies. This review also emphasizes key gaps in the literature, including the underrepresentation of female athletes, the lack of diagnostic biomarkers for living individuals, and the need for standardized preclinical models. Addressing these gaps is essential to improving diagnosis, treatment, and overall patient outcomes.

A version of this paper was previously posted to the Preprints.org server on February 25, 2025 [17].

## Review

Clinical presentation in athletes

Examining the clinical presentation of CTE in athletes can help us better understand the signs, severity, and outcomes of this debilitating disease. While this disease cannot be diagnosed while alive [18], there are common symptoms associated with CTE, such as memory impairment, confusion, mood disturbances, dementia, and motor dysfunction [9,19]. Furthermore, the occurrence and prognosis of CTE are unique to the sport that caused it [6,20,21].

The clinical presentation of CTE is severe in NFL athletes [11]. This is due to the high frequency and severity of head impacts they face, as well as the duration of exposure starting as early as adolescence [22]. This, in turn, has produced severe symptoms such as advanced dementia and more intense behavioral changes compared to their soccer and boxing counterparts [4,23]. However, the severity and progression of CTE are due to differences in impact mechanisms. Studies indicate that NFL players experience greater linear acceleration forces from helmet-to-helmet and helmet-to-ground collisions, whereas boxers sustain higher rotational forces from punches [24,25]. These differences contribute to distinct patterns in the types of symptoms observed in each sport [26]. Moreover, football positions influence the incidence rates of mTBI. Running backs and wide receivers, who experience more frequent head impacts, suffer from a higher rate of mTBI than linemen [27]. Linemen, who generally have stronger necks and larger girths, experience a reduction in the linear accelerations of their heads compared to running backs, which lowers their risk of getting a concussion [26]. These positional differences can partially explain the varying severity of CTE in football athletes. Furthermore, it is essential to consider that linemen often experience more sub-concussive helmet-to-helmet impacts, while wide receivers are exposed to more intense impacts from tackles, which may explain the differences in clinical presentations of CTE [26].

Beyond these sport- and position-specific differences, CTE tends to follow a progressive course that begins in midlife, often after retirement from sport [19]. Early symptoms typically involve behavioral and psychiatric changes such as irritability, anger, apathy, and increased suicidality, as reported by families and caregivers [19]. In some cases, athletes have also exhibited trauma-related symptoms such as hypervigilance, flashbacks, or intrusive thoughts, which can resemble features of post-traumatic stress disorder (PTSD) [9]. As the disease progresses, cognitive impairments, particularly in episodic memory and executive functioning, become more pronounced. In later stages, movement abnormalities, speech difficulties, and ocular changes may also emerge. While dementia can occur, it is relatively uncommon, possibly because many individuals with CTE die prematurely due to suicide, overdose, or accidents [19]. Symptoms generally appear years after retirement, with a mean age of onset around 42.8 years and an average latency of eight years post-retirement [19]. The disease follows an insidious and gradual course, with some athletes becoming symptomatic even before retiring. Notably, duration varies by sport; boxers tend to experience a longer course, averaging 20 years, whereas American football players average around six years [19].

In addition to sport-related factors, sex-based differences in symptom expression have also been observed in other forms of TBI and may be relevant in CTE. Some evidence suggests that female athletes report more severe post-concussive symptoms compared to their male counterparts [8]. However, the underrepresentation of women in CTE research has limited our understanding of how clinical presentation may differ by sex. Additional studies are needed to determine whether biological sex influences the onset, progression, or severity of CTE symptoms.

While the clinical presentation of CTE in NFL athletes can vary in severity depending on factors such as position and impact type, it is generally severe in athletes who experience high-frequency and high-intensity head impacts. In contrast, the clinical presentation in soccer athletes is often more evasive [28]. For high school sports, the frequency of concussions in soccer was seen at a rate much lower than in American football [28]. American football constituted 47.1% of all concussions, and girls’ soccer constituted over 8% of all concussions [28]. Head contact in soccer is also limited to headers, which at the professional level is done on average 12 times a game [29], and at the youth level is done on average once a game [30]. However, if the soccer ball has a pressure that is too high and/or becomes waterlogged, the chances of head trauma dramatically increase [29]. Due to this, the presentation of CTE in soccer athletes still exists at a mild level. For example, a team of Norwegian football players displayed mild to severe deficits in attention, concentration, memory, and judgment, reflecting the presentation that soccer athletes exhibit when dealing with CTE [23]. An investigation done on a Dutch Soccer Club took this further, identifying the effect of soccer positions on CTE. After comparing the Dutch Club to a control group of 27 elite noncontact sport athletes, they determined a general presentation consisting of impaired memory, planning, and visuoperceptual processing [31]. They also concluded that forward and defensive positions exhibited more impairment due to their higher likelihood of heading the ball [31]. Implementing better rules and protocols, such as reducing ball pressure, will cause CTE cases in soccer players to decrease [29]. For this reason, CTE does not have to be a prevalent issue for soccer players in the future.

Research on CTE initially began with boxing, where it was originally referred to as punch drunk syndrome. A report in 1928 outlined this syndrome, recognizing boxers who were described by fans to be eccentric or erratic. Boxers exhibiting these cuckoo behaviors were known to have sustained significant head trauma [4,32]. A study was conducted on 23 boxers who shared presentations of minute changes in gait or staggered walks back to their corners between rounds [33]. This study also noted that the syndrome had severe cases where presentation also included Parkinsonian gait, tremulousness, vertigo, and cognitive symptoms such as mental deterioration [33]. The presentation of CTE in boxers depends on their style. Boxers who displayed a grit and grind style showed their symptoms on stage, whereas boxers who utilize footwork and speed may avoid symptoms altogether [4]. The autopsies of 309 traumatic cerebral hemorrhage patients give us a glimpse into the late-life prognosis of these boxers ridiculed with CTE. Out of these 309 autopsies, nine cases were not associated with any signs of overt cortical injuries or skull fractures. These nine cases came from boxers who had sustained excessive head trauma and were labeled as punch drunk [4,33]. This study shows us that the effects of CTE follow boxers into their late lives.

While the presentation of CTE in football, soccer, and boxing all share a commonality in "mental deterioration," they are all also unique in their own ways. The severity of CTE in NFL athletes is unmatched due to the repeated head trauma they endure on every play. Soccer athletes show a mild presentation of CTE because of their infrequent use of their heads in their sport. Boxers can show symptoms of CTE even while on stage fighting. Further examination of these symptoms will allow us to better understand CTE and the signs, severity, and outcomes of this debilitating disease.

Neurobehavioral and neurological mechanisms

Understanding the neurobiological and neurological mechanisms causing CTE is important for developing effective treatments and preventive strategies. Repeated brain injuries lead to the accumulation of the phosphorylated tau protein (p-tau), a hallmark of CTE [18]. The protein forms neurofibrillary tangles, astrocytic tangles, and tau-positive neurites, often clustered around small blood vessels in the cortical sulci [18,34]. Additionally, in the early or mild stages of CTE, observable brain changes are often minimal but may include the cavum septum pellucidum, slight enlargement of the frontal and temporal horns of the lateral ventricles, and prominent perivascular spaces, particularly in the temporal lobe's white matter [34].

CTE is a distinctive tauopathy characterized by neurodegeneration that progresses through four stages [34]. In stage 1, the brain appears normal, with p-tau accumulation limited to certain areas, such as the lateral and frontal cortices, and proximal to small blood vessels in the depth of sulci [35-37]. By stage 2, localized macroscopic abnormalities become apparent [35,36]. In stage 3, there is a global reduction in brain weight, with mild frontal and temporal lobe atrophy and ventricular dilation [35,36]. Stage 4, the most severe stage, is marked by profound brain atrophy, particularly in the frontal and medial temporal lobes, with brain weights as low as 1,000 grams reported [35,36].

Along with tau protein accumulation, individuals with CTE may also show the buildup of phosphorylated transactive response DNA-binding protein 43 (p-TDP-43) in both the white matter and cortical regions [9,37,38]. This protein appears as neuritic accumulations and inclusions within neurons and glial cells [9,39-41]. The National Institute of Neurological Disorders and Stroke and the National Institute of Biomedical Imaging and Bioengineering (NINDS-NIBIB) panel identified TDP-43 pathology as a feature of CTE and recognized its role as a criterion for diagnosis post-mortem [42].

Pathological investigations have shown that repeated brain injuries can trigger chronic neuroinflammation, which may contribute to neuronal damage and the progression of CTE [43-46]. Neuroinflammation is also linked to more extensive p-tau pathology in CTE [39]. The significant role of neuroinflammation in the development and progression of CTE suggests that inflammatory markers could be valuable for future diagnosis and possible treatment [43,47].

Additionally, targeting these inflammatory pathways may offer new opportunities for managing CTE. Research from the DIAGNOSE CTE Project [43] found that cerebrospinal fluid (CSF) analysis in former football players with neurobehavioral dysregulation showed a 21.8% increase in interleukin-6 (IL-6) levels compared to controls (1.73 pg/mL vs. 1.42 pg/mL, p = 0.032), along with elevated C-reactive protein (CRP) [43]. IL-6 levels were significantly associated with greater emotional dyscontrol, affect lability, and impulsivity, reinforcing its role in neurobehavioral symptoms. However, IL-6 levels in unexposed individuals were not significantly different (p = 0.071), suggesting an overlap that may limit its clinical utility [43]. Additionally, plasma neurofilament light (NfL), a marker of neurodegeneration, was linked to executive dysfunction and processing speed deficits in older former players, highlighting the interplay between inflammation and neurodegeneration in CTE pathology [43].

Diagnosis of CTE

Despite advancements in understanding CTE's mechanisms, diagnosing CTE remains challenging. Currently, there is no definitive method for diagnosing CTE in living individuals [18].

Current efforts to isolate CTE biomarkers in CSF rely on the known mechanisms of cerebral ventricular injury associated with mTBI [2,48]. These mechanisms alter the composition of CSF, but the exact reasons for elevated CSF biomarkers in CTE are not fully understood [26,48]. Elevated CSF biomarkers, such as tau, neurofilament protein, glial (fibrillar astrocytic) protein (GFAP), and S100β, may persist for extended periods after repetitive mTBI. While some biomarkers, like p-tau, return to baseline within two weeks, others, such as NfL polypeptide, remain elevated for longer durations, though the exact timeframe is still under investigation [26]. Although several studies have explored fluid biomarkers in both CSF and serum following TBI and blast injuries, their diagnostic utility for CTE remains limited. Biomarker levels can appear normal even in individuals with known exposure to high-impact blast, possibly due to the timing of sampling or rapid clearance of these markers from circulation [48]. This discrepancy between postmortem neuropathological findings and acute-phase biomarker data underscores a critical gap: no fluid biomarker has yet been validated for chronic, long-term detection of CTE in living individuals [48]. Moreover, many of these studies focus on acute injury rather than persistent neurodegenerative processes, making it unclear whether current biomarkers can reflect the unique pathophysiology of CTE [48]. Further research is necessary to identify chronic-stage, disease-specific biomarkers that could help diagnose CTE earlier and more reliably.

Imaging techniques such as computed tomography (CT) and magnetic resonance imaging (MRI) are commonly used to evaluate mTBI but often fail to definitively reveal brain injury associated with CTE [48]. Positron emission tomography (PET) imaging may help differentiate CTE from other neurodegenerative diseases by detecting beta-amyloid, but it remains an experimental tool rather than a definitive diagnostic method [26,49].

Currently, the most accurate diagnosis of CTE is made through postmortem neuropathological analysis, where the brain is examined microscopically after death [18,35]. While clinical assessments can help determine a likely diagnosis based on symptoms and history of head trauma, most current exams and imaging techniques primarily serve to rule out other conditions rather than confirm CTE [18]. Moreover, most available diagnostic data is based on male subjects, with limited research on how CTE is present in women [37]. The overwhelming focus on male athletes in these studies means there is little understanding of whether the disease manifests differently in women [37]. Without targeted research on female populations, it remains unclear whether gender-based differences exist in biomarker expression, symptom progression, or imaging findings, potentially leading to misdiagnosis or underdiagnosis in women. Addressing this gap is crucial for developing more accurate and inclusive diagnostic criteria.

Treatment and prevention of CTE

Supportive interventions, including cognitive rehabilitation, motor therapy, mood and behavior therapy, aerobic exercise, a healthy diet, and mindfulness practices, aim to mitigate symptoms and improve patients’ quality of life [35,50]. Cognitive rehabilitation, in particular, helps individuals enhance memory, decision-making, and overall cognitive functioning following traumatic brain injury [51]. This approach involves continuous memory review to strengthen cognitive abilities, guiding the brain to recall past knowledge, overcome emotional barriers, and identify personal strengths and weaknesses after injury. It also improves attention, a fundamental cognitive skill supporting other mental processes, which in turn aids motor recovery, crucial for rehabilitation [51]. Given that repetitive concussions and blows in sports contribute significantly to CTE development [52,53], prevention strategies are essential to reduce risk. These include rule changes, stricter regulations, and the use of safer head equipment such as helmets [52,54]. While helmets have effectively lowered severe injuries, they may offer insufficient protection against lower-force impacts that are strongly implicated in concussions, especially in athletes [52]. A thorough review of helmet design evolution and regulatory standards such as those established by the National Operating Committee on Standards for Athletic Equipment (NOCSAE) reveals a substantial decrease in fatalities (by 74%) and serious head injuries (from 4.25 to 0.68 per 100,000) since their implementation [55]. Both the materials used and the protective design of helmets are critical for injury prevention, and competition among manufacturers continues to drive safety innovations. Continuous advancements in helmet technology remain necessary to improve protection against all types of head impacts and better prevent CTE [56-58].

CTE in rodents

Rodent models have been widely developed to study the mechanical forces underlying concussions and their long-term effects, particularly in the context of CTE [59]. Various models, including controlled cortical impact (CCI), fluid percussion (FP), and weight drop, have been used to simulate repeated mTBI and its potential role in CTE pathogenesis [58-65].

The CCI model is one of the most commonly utilized paradigms for studying brain injury in animals [58,66]. It provides precise control over biomechanical parameters such as impact force, velocity, and tissue deformation, allowing researchers to replicate aspects of TBI [66]. However, to more accurately simulate concussive injuries, modifications have been made to eliminate the need for craniotomy. These adaptations include using rubber or silicone tips to impact the skull directly or employing a form-fitting steel cap to prevent focal necrotic lesions [59]. Research has shown that avoiding craniotomy produces a more clinically relevant injury, as direct impact on the dura rather than the skull leads to significant cortical tissue loss and hematoma formation [58-68].

Studies using the modified CCI model have demonstrated that varying impact parameters can influence injury severity and outcomes. The impact parameters used in studies modeling repeated concussions range from over 3 to 6 m/s for acceleration and from 31.5 to 500 ms for dwell time [59]. However, many studies lack objective measures of injury severity, such as the presence of an apneic period or the loss of the righting reflex, making it difficult to compare results across different models [58-60].

Despite these limitations, rodent models have successfully replicated several features of CTE, including long-term behavioral and neuropathological changes [59-61]. Some models examine injury effects over extended periods, up to a year post-injury, providing insights into the progressive nature of CTE pathology [69,70]. However, challenges remain in translating these findings to humans, as rodent lifespans differ significantly from human lifespans, potentially affecting the disease's time course. Nonetheless, these models are crucial for investigating the long-term consequences of repeated head trauma and understanding the underlying mechanisms of neurodegeneration.

The FP model, another widely used approach, has been modified to produce less severe injuries by reducing the force of the fluid pulse [58,71]. Unlike CCI, FP delivered injury through intact dura, generating brain displacement and deformation via a craniotomy and fluid pressure pulse [58-74]. Studies using repeated FP injuries have demonstrated cognitive deficits in both the short and long term, similar to findings from modified CCI models [71,72]. However, FP-induced injuries often result in more severe motor impairments, likely due to increased cortical damage [58].

Repetitive brain trauma has been linked to neuropathological changes consistent with CTE, including axonal degeneration and neuroinflammation [69,70]. In athletes, repeated concussions can initiate long-term neurodegenerative processes such as dementia pugilistica and CTE [69,70,74]. Closed-head injury models, which apply force directly to the intact skull, better mimic real-world concussive injuries by allowing unrestricted head movement, including lateral and rotational forces [75]. These models produce diffuse brain injuries without the cortical contusions or hemorrhages seen in CCI and FP models [58,75]. Research using the weight-drop model has demonstrated cognitive deficits persisting up to a year post-injury, along with delayed motor impairment and depressive behaviors following repeated impacts over six weeks [58-62]. These findings suggest that early behavioral symptoms may serve as indicators of chronic neuropathology associated with repeated head trauma [58,70,75].

Comparisons across studies remain challenging due to variations in experimental parameters. When examining CTE in retired athletes, confounding factors such as alcohol and drug use must be considered, as studies suggest that retired athletes, particularly former soccer, rugby, hockey, and NFL players, have higher rates of alcohol misuse, which may influence brain volume and pathology [19]. While post-mortem human studies remain essential for understanding CTE pathology, rodent models offer a controlled environment to study the direct effects of repeated mild TBI, free from lifestyle-related confounds [76]. These models continue to provide valuable insights into the mechanisms linking RHIs to neurodegeneration and the development of CTE [58].

Discussion

Most CTE studies have focused on male athletes, leading to a lack of understanding of how sex-specific factors may influence the development, progression, and diagnosis of the disease. Historically, there have been very few documented cases of CTE in women. Two earlier cases involved non-athletes: one was a 76-year-old woman who suffered years of domestic abuse [77]. Postmortem examination revealed abnormal thickening of her ears, resembling the cauliflower ears seen in boxers, a clear sign of repeated blunt force trauma. Her brain pathology closely resembled that of dementia pugilistica, the term historically used for CTE in boxers, with extensive neurofibrillary tangles and diffuse plaques in the frontal cortex. The study’s authors concluded that repeated head trauma was a significant contributing factor to her neurodegeneration [77]. The second case involved a 24-year-old autistic woman who engaged in chronic self-injury behaviors, including head-banging, eye-gouging, and self-biting, from childhood [78]. Despite multiple therapeutic interventions, her self-injurious behaviors persisted, leading to significant neurodegeneration. At autopsy, her brain exhibited neurofibrillary tangles in the perirhinal and entorhinal cortex, similar to findings in individuals with a history of repeated head trauma, suggesting that the mechanical forces from her self-inflicted injuries contributed to the CTE pathology [78]. More recently, a case was reported in a former professional female footballer in her late 20s, who died by suspected suicide after an 18-year history of contact sports participation [8]. This is the first documented case of CTE in a professional female athlete. The absence of overt clinical symptoms before her death underscores the possibility that CTE may present differently in women or go unrecognized.

Although research has traditionally concentrated on male athletes, recent studies suggest sex-based differences may affect how CTE develops. Hormonal factors in women, such as the neuroprotective effects of estrogen, may influence CTE development and progression, with a drop in estrogen levels after menopause potentially affecting the brain’s response to RHIs [79]. Additionally, the menstrual cycle could impact how women experience CTE symptoms, though more research is needed to fully understand this connection [79]. Social and cultural dynamics also play a role: female athletes may underreport symptoms, face underdiagnosis, or receive treatments based on male-centered research paradigms [80]. As a result, the medical community may overlook important sex-specific symptoms and progression patterns.

Supporting this, one study using male and female rodents found differences in acute biomarkers and tau aggregation patterns following repeated mTBI. Males were more prone to behavioral changes like anxiety, while females showed early changes in neuron-specific enolase levels [81]. Additionally, male rodents exhibited significantly increased tau aggregation in the brain at 12 weeks post-injury, further supporting the hypothesis that CTE may manifest differently between sexes [81]. These findings highlight biological sex as a potentially critical factor in CTE progression, diagnosis, and symptom expression.

While standard imaging techniques such as MRI and PET remain inconclusive for definitively diagnosing CTE, recent innovations could expand the potential of these tools to detect disease-related pathology in living individuals. Techniques like diffusion tensor imaging (DTI) and neurite orientation dispersion and density imaging (NODDI) allow researchers to detect subtle changes in white matter structure and axonal integrity that may be linked to repeated head trauma [82]. Also, tau-targeting PET scans using radiotracers such as fluorine-18-labeled FDDNP (2-(1-{6-[(2-[^18^F]fluoroethyl)(methyl)amino]-2-naphthyl}ethylidene)malononitrile) and flortaucipir have demonstrated binding patterns consistent with postmortem findings of tau protein aggregation [83]. However, these tools require validation through longitudinal studies comparing imaging data with postmortem findings [83,84]. Currently, CTE diagnosis remains limited to autopsy, and the lack of in vivo diagnostic tools continues to hinder early intervention.

Without more standardized diagnostic criteria and prospective research, the true prevalence of CTE, especially in underrepresented populations, remains unknown. While animal models and retrospective studies offer important insights, there is an urgent need for more inclusive human research, particularly involving female athletes and nontraditional populations [85].

## Conclusions

This focused review highlights the urgent need for a deeper understanding of CTE to enhance early diagnosis, intervention, and long-term management. Given the progressive and irreversible nature of CTE, identifying at-risk individuals, particularly athletes, remains a major challenge. Although current diagnostic methods largely rely on postmortem neuropathological examination, recent research advances show promise in identifying biomarkers and potential early detection methods. The accumulation of hyperphosphorylated tau protein plays a key role in neurodegeneration, contributing to cognitive and behavioral disturbances. Despite the emphasis on tau pathology, the exact mechanisms behind neuronal damage remain unclear, underscoring the need for targeted therapeutic strategies to slow or halt disease progression.

To ensure a more inclusive understanding of CTE, future research must prioritize the inclusion of female participants and diverse populations. Variability in imaging techniques and inconsistent methodologies across studies also complicate the interpretation of results, making it difficult to draw definitive conclusions. Standardizing imaging procedures and adopting consistent diagnostic criteria will enhance the reliability and comparability of findings. Additionally, longitudinal studies are essential for tracking the progression of CTE over time. Addressing these research gaps will not only improve diagnostic and treatment approaches but also aid in the development of effective prevention strategies for this debilitating condition.
